# Influence of *Apis mellifera syriaca* Bee Venom on Nociception and Inflammatory Cytokine Profiles in Experimental Hyperalgesia

**DOI:** 10.3390/toxins17010018

**Published:** 2025-01-01

**Authors:** Mohamad Ayoub, Salma Fayjaloun, Rabih Roufayel, Dany El Obeid, Ziad Fajloun, Mohamad Rima, Marc Karam

**Affiliations:** 1 Faculty of Sciences, University of Balamand, Al-Kourah, P.O. Box 100, Tripoli 1300, Lebanon; mohamad.b.ayoub@std.balamand.edu.lb; 2 Department of Cell Culture, Laboratory of Applied Biotechnology (LBA3B), Azm Center for Research in Biotechnology and Its Applications, EDST, Lebanese University, Tripoli 1300, Lebanon; salma_fayjaloun@hotmail.com; 3 College of Engineering and Technology, American University of the Middle East, Egaila 54200, Kuwait; rabih.roufayel@aum.edu.kw; 4 Faculty of Agriculture & Veterinary Sciences, Lebanese University, Dekwaneh, Beirut 2832, Lebanon; dany.elobeid@ul.edu.lb; 5 Faculty of Sciences 3, Lebanese University, Michel Slayman Tripoli Campus, Ras Maska 1352, Lebanon; 6 Department of Natural Sciences, Lebanese American University, Byblos P.O. Box 36, Lebanon

**Keywords:** *A. mellifera syriaca*, bee venom, hyperalgesia, inflammation, immunomodulatory activity, cytokines, pain

## Abstract

Hyperalgesia is a condition marked by an abnormal increase in pain sensitivity, often occurring in response to tissue injury, inflammation, or prolonged exposure to certain medications. Inflammatory mediators, such as cytokines IL-1β, IL-6, and TNF-α, play a central role in this process, amplifying pain perception. Developing effective treatments that address the underlying mechanisms of hyperalgesia is an active field of research. *Apis mellifera syriaca* venom demonstrated potential immunomodulatory activity associated with cytokine release in vivo. Therefore, the aim of this study is to evaluate the effect of *Apis mellifera syriaca* bee venom (*Ams*BV) on pain sensitivity in a formalin-induced hyperalgesia mice model and to evaluate the potential role of cytokines associated with the nociception of pain. The hotplate test, used to measure pain latency, showed that hypersensitivity to pain was induced in formalin-injected male mice only, with no changes in females, suggesting a sex-based response to formalin. When applied, *Ams*BV reduced pain sensitivity in males, suggesting pain relief potential. At the molecular level, *Ams*BV was able to reduce pro-inflammatory interleukin IL-4 and cytokine IFN-γ, emphasizing its immunomodulatory potential. Interestingly, the venom restored anti-inflammatory IL-10 levels that were significantly decreased in hyperalgesia males. Together, these findings highlight the therapeutic potential for *Ams*BV in managing inflammation and reducing pain, particularly hyperalgesia.

## 1. Introduction

Hyperalgesia refers to an increased sensitivity to pain, often resulting from damage or overstimulation of the nerves involved in pain transmission [1]. It can be classified into two main types: opioid-induced and injury-induced hyperalgesia. Opioid-induced hyperalgesia (OIH) occurs when high doses of opioids, such as morphine, paradoxically increase sensitivity to pain, while injury-induced hyperalgesia develops from nerve or tissue damage [2]. Pain begins when nociceptors, specialized neurons in the somatosensory system, detect harmful stimuli. These nociceptors are equipped with molecular sensors like transient receptor potential (TRP) channels and G-protein coupled receptors (GPCRs), which detect various types of damage [3]. Inflammatory responses following tissue injury trigger the release of cytokines, which increase nociceptor sensitivity [4]. Research has demonstrated that cytokine and chemokine signaling pathways influence pain-like behavior in animal models of inflammation and neuropathy [5]. Key cytokines such as IL-1β, IL-6, and TNF-α play significant roles in enhancing pain perception and hyperalgesia [6]. IL-1β, in particular, is a major contributor to mechanical hyperalgesia, while IL-6 and TNF-α also promote pain through similar pathways [7,8]. Contrarily, cytokines like IL-4 and IL-10 have been found to suppress cytokine-mediated inflammatory hyperalgesia [9]. Understanding the molecular pattern of inflammation-induced hyperalgesia can facilitate screening for drugs/natural compounds that can modulate pain.

Honeybee venom (BV) from *Apis mellifera* is a complex mixture of biologically active substances that has been widely used in traditional medicine and extensively studied for its therapeutic properties [8,9]. *A. mellifera* BV primarily consists of proteins and peptides, with melittin being the most abundant and well-researched component, followed by phospholipase A2 (PLA2), an enzyme known for its allergenic potential alongside histamine [8,9]. Melittin, comprising 40–60% of the dry venom, has been shown to induce pain and inflammation at high concentrations, but at lower doses, it exhibits potent anti-inflammatory effects [10,11,12]. Studies have demonstrated that *A. mellifera* BV inhibits the production of key inflammatory cytokines such as IL-1β, IL-6, IL-8, TNF-α, and IFN-γ, suggesting its potential for modulating pain and inflammation [13,14]. Therefore, *A. mellifera* BV was proposed as an effective anti-nociceptive treatment for hyperalgesia [15].

The venom of the Middle Eastern bee, *Apis mellifera syriaca*, has been the subject of several studies that investigated its proteomic content and biological properties. This venom (named *Ams*BV here) has some significant biological properties with pharmaceutical relevance, including anticoagulant [16], antibacterial [17], anti-cancer [18,19], and anti-inflammatory activities [20,21]. The full panel of *Ams*BV biological activities is still under investigation, and will definitely extend given the diverse pathways in which bee venoms can interfere. The anti-inflammatory properties of *A. mellifera* BV, in general, have been investigated in various conditions, including acne vulgaris, neuroinflammation, amyotrophic lateral sclerosis, atherosclerosis, arthritis, and liver inflammation [21]. Similar studies have demonstrated that *Ams*BV exhibits anti-inflammatory effects by decreasing IFN-γ, TNF-α, IL-4, and IL-10 [22]. However, the effects of *Ams*BV on cytokine-mediated inflammatory hyperalgesia, particularly following formalin-induced pain, remain unexplored.

Despite limited research on the use of BV for hyperalgesia treatment, its ability to neutralize the pro-inflammatory effects of cytokines suggests a promising therapeutic potential. In this study, we aim to evaluate the effects of *Ams*BV on hyperalgesia, focusing on its influence on physiological pain pathways and cytokine expression.

## 2. Results

### 2.1. The Effect of Apis mellifera syriaca Bee Venom (AmsBV) on Pain Sensitivity in Hyperalgesia

**Figure 1:**
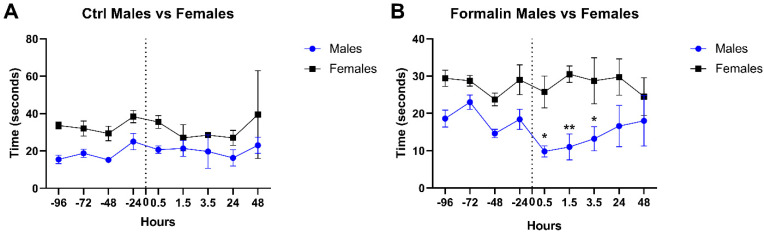
Validation of formalin-induced hyperalgesia in mice models. Changes in pain sensitivity were reported using the hotplate test. Male and female mice were injected with formalin or an equivalent volume of PBS (at T = 0) after recording baseline sensitivity daily over 4 days. Pain sensitivity was tracked up to 2 days post-formalin injection. * p ≤ 0.05; ** p ≤ 0.01.

In control groups of mice, the pain threshold, assessed by the time needed for the animal to retract its paw from the hotplate, was stable before (during baseline recordings) and after injection with no significant difference in both males and females (Figure 1A). Interestingly, all over the analysis, the pain threshold was higher in females compared to males, where female mice were significantly more tolerant to pain compared to male mice. This finding is consistent with sex-related differences in response to pain [23]. Upon formalin injection, the reaction time of male mice was significantly decreased 30 min post-formalin injection, highlighting an increased sensitivity to pain triggered by formalin. This increased sensitivity to pain remained significantly detectable until 3.5 h post-formalin injection (Figure 1B). This result suggests that formalin lowers the pain threshold in males, but not in females, validating the increase in sensitivity that mimics hyperalgesia.

**Figure 2:**
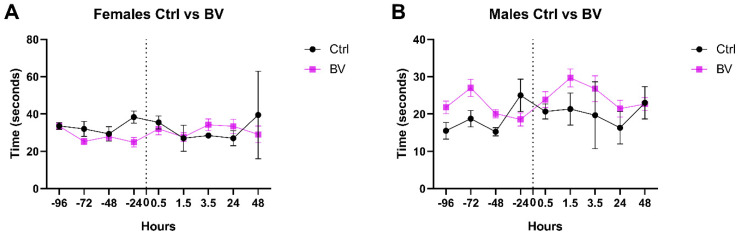
*Ams*BV does not affect pain threshold in mice. Alteration in pain sensitivity in male and female mice injected with *Ams*BV or an equivalent volume of PBS (at T = 0), after recording baseline sensitivity daily over 4 days, was investigated up to 2 days post-formalin injection.

The consequence of *Ams*BV on pain sensitivity was also checked using the hotplate test for a duration of 48 h after injection. Both sexes were insensitive to *Ams*BV and did not show any significant change after *Ams*BV was injected (Figure 2A,B). These results suggest that venom cannot modulate or affect the pain sensitivity threshold in normal conditions.

**Figure 3:**
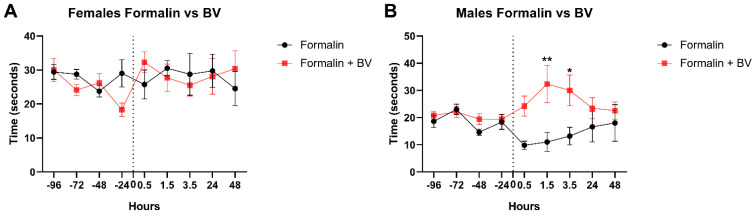
*Ams*BV decreases pain sensitivity in formalin-induced hyperalgesia in male mice models. Male and female mice were injected with formalin or an equivalent volume of PBS (at T = 0) after recording baseline sensitivity daily over 4 days. BV was injected 10 min post-formalin injection and pain sensitivity was tracked up to 2 days using the hotplate test. * p ≤ 0.05; ** p ≤ 0.01.

*Ams*BV was not able to increase the pain threshold in formalin-injected females as the time required to retract their paws remained comparable to the control group (Figure 3A). However, *Ams*BV was able to increase the pain threshold in a formalin-injected mouse model as seen with the significant increase detected at 1.5 h post-formalin injection (Figure 3B). This significant increase remained detectable even at 3.5 h and faded out at 24 and 48 h (Figure 3B), which was synchronized with the fading out of the hypersensitivity to pain seen in Figure 1B. Together, these findings suggest that *Ams*BV is able to decrease pain sensitivity in formalin-induced hyperalgesia seen in males only.

### 2.2. The Effect of AmsBV on IL-4 and IL-6 Levels in the Spleens of Formalin-Induced Hyperalgesia Mice Model

**Figure 4:**
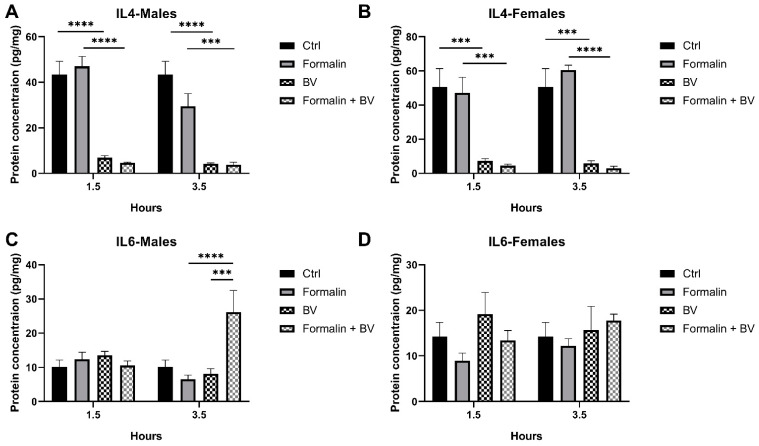
Evaluation of interleukin-level changes upon *Ams*BV in formalin-induced hyperalgesia mice model. (**A**) IL-4 levels were investigated in male and (**B**) female mice. (**C**) IL-6 levels were quantified in male and (**D**) female mice following venom injection with/without formalin preinjection. Values are means ± SEM for *n* = 5 per group. *** p ≤ 0.001; **** p ≤ 0.0001.

Since pro- and anti-inflammatory interleukins are increasingly recognized for their complex role in modulating pain and inflammation, the levels of interleukins were quantified and are reported in Figure 4. Following subcutaneous injections of mice with *Ams*BV, IL-4 levels significantly dropped in both sexes as compared to their respective controls (*p* < 0.0001). In a formalin-preinjected mouse model, *Ams*BV was also able to induce a significant decrease in IL-4 levels in both sexes that was comparable to that observed in mice receiving BV alone (Figure 4A,B).

Of note, formalin injection did not affect IL-4 levels at any of the time points studied (Figure 4A,B), suggesting that IL-4 levels are not affected in the formalin-induced hyperalgesia mice model. Also, IL-6 levels remained unchanged upon formalin or *Ams*BV injections at the two timepoints studied (Figure 4C,D), suggesting that IL-4 levels are not affected in the formalin-induced hyperalgesia mice model. However, in the formalin-preinjected mice, *Ams*BV significantly increased IL-6 levels in males 3.5 h post injection (Figure 4C). The contradictory pro- and anti-inflammatory roles of IL-6 prevents the conclusion of its implication in pain; however, these results align with the immunomodulatory effects of *Ams*BV [24].

### 2.3. The Effect of AmsBV on the Levels of IL-10 and IFN-γ in the Spleens of Formalin-Induced Hyperalgesia Mice Model

**Figure 5:**
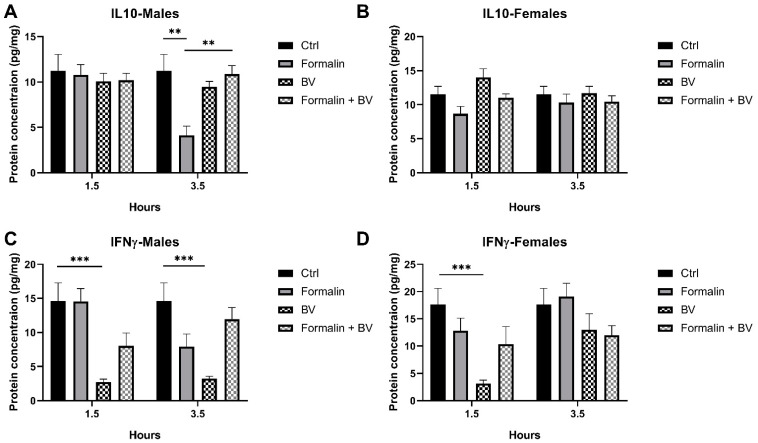
Evaluation of interleukin and cytokine-level changes upon *Ams*BV in formalin-induced hyperalgesia mice model. (**A**) IL-10 levels were investigated in male and (**B**) female mice. (**C**) IFN-γ levels were quantified in male and (**D**) female mice following venom injection with/without formalin preinjection. Values are means ± SEM for *n* = 5 per group. ** p ≤ 0.01; *** p ≤ 0.001.

To further investigate interleukin levels in formalin-induced hyperalgesia mice, IL-10 and IFN-γ levels, known to play important and controversial roles, respectively, in pain sensitization were investigated [25]. Interestingly, IL-10 levels were significantly reduced in formalin-injected mice, compared to their control (*p* < 0.01) (Figure 5A). These results suggest that formalin reduces IL-10 levels in mice, emphasizing the link between inflammation and hyperalgesia. In contrast, the venom alone was not able to induce changes in IL-10 expression levels; however, it was able to significantly restore IL-10 levels in formalin-preinjected male mice to normal levels reported in control mice (*p* < 0.01) (Figure 5A). Female mice did not show any significant changes in IL-10 levels across condition groups (Figure 5B).

Given the reducing effect of IL-10 on inflammatory hyperalgesia, our results suggest that *Ams*BV reduces pain sensitivity by restoring IL-10 levels in formalin-induced hyperalgesia. In addition, BV downregulated IFN-γ expression in both sexes when injected alone (Figure 5C,D); however, in formalin-preinjected mice, the venom did not show any significant change in IFN-γ levels (Figure 5C,D). Together, these findings suggest that IFN-γ expression is not changing in the formalin-induced hyperalgesia mice model and that *Ams*BV can modulate IFN-γ levels.

## 3. Discussion

Characterized by increased sensitivity to feeling pain and an extreme response to pain, hyperalgesia may occur when there is damage to the nerves or chemical changes to the nerve pathways involved in sensing pain. Inflammatory hyperalgesia is mediated by two main pathways, with sympathetic amines and/or prostanoids acting as the final mediators [26]. Formalin, a known inducer of inflammation and spinal sensitization, increases the release of prostaglandins and nitric oxide in the spinal cord [27]. Subcutaneous formalin injection was widely used as a nociceptive stimulus in rodents to study hyperalgesia [28]. The hypothetical explanation of formalin-induced hyperalgesia is the capability of causing an inflammatory response by triggering a local (site of injection) inflammatory reaction, by activating pain pathways, hormonal response, and neurological effects. Given the tight link between hyperalgesia and inflammation, it was thought that regulating the inflammatory pathway may help in reducing increased pain sensitivity.

In addition to several biological activities, the immunomodulatory effects of *A. mellifera syriaca* bee venom (*Ams*BV) in mice were reported as the venom was able to modulate the expression of pro- and anti-inflammatory interleukins and cytokines. Many other studies have focused on identifying, characterizing, and purifying proteins and enzymes within *Apis mellifera* bee venom (*Am*BV), while others have highlighted its biological properties in vitro [10,11,21]. The modulation of inflammation by *Am*BV was in part assigned to melittin, a major component of the venom, which has been shown to exhibit anti-inflammatory activity in vitro [20]. This protein is also part of the complex mixture of compounds found in the venom of *A. mellifera syriaca* [19].

These findings combined with the inflammation-based mechanism of hyperalgesia shaped our study, aiming to further understand the potential of *Ams*BV in modulating inflammation linked to pain sensitivity as the anti-hyperalgesia agent in induced inflammatory pain. To this end, behavioral and biochemical assessments of hyperalgesia were studied in a formalin-induced hyperalgesia mouse model after being injected with *Ams*BV. First, we validated the reliability of our hyperalgesia model by checking the changes in pain sensitivity in mice. Interestingly, formalin increased the pain sensitivity of male mice only, without affecting female mice, which might be related to hormonal fluctuations that modify inflammatory responses. When injected alone, *Ams*BV did not affect pain sensitivity, suggesting that the venom cannot induce changes in pain threshold sensitivity. This hypothesis is strengthened by the fact that in females, which were unsensitive to formalin injection, the venom injection had no effect on pain sensitivity. However, when injected in formalin-preinjected male mice, the venom was able to significantly decrease the pain sensitivity witnessed by the increased time needed for mice to retract their paws from the hotplate. Together, these findings suggest that *Ams*BV can modulate pain sensitivity in hyperalgesia.

Since hyperalgesia is commonly observed in areas of tissue damage or inflammation, and *Ams*BV can modulate inflammation, molecular analysis was conducted to quantify interleukin and cytokine levels in formalin-injected mice, in the presence and absence of *Ams*BV. First, we started investigating whether formalin-induced hyperalgesia observed in male mice can translate into changes in interleukin and cytokines levels. Among all tested molecules, only IL-10 was significantly decreased in the formalin-injected male group. This finding is consistent with previous research highlighting the beneficial role this anti-inflammatory interleukin plays in treating hyperalgesia [9,29,,30]. *Ams*BV was able to restore IL-10 levels to normal levels in formalin-injected male mice, which might explain the decrease in sensitivity to pain induced by formalin. Hence, female formalin-injected mice not showing changes in pain sensitivity did not show any changes in IL-10 levels when injected with *Ams*BV. Our findings suggest that *Ams*BV modulates pain sensitivity in hyperalgesia by regulating IL-10 levels. These findings align with the immunomodulatory potential of *Ams*BV that may be beneficial in treating inflammation-related diseases.

On the other hand, mice receiving only *Ams*BV witnessed a significant decrease in IL-4 and IFN-γ in both in females in males. However, IL-6 levels and IL-10 were not affected by BV injection, suggesting that *Ams*BV regulates cytokine and interleukin levels in a similar fashion, with no differences in response based on sex. These findings align with our previous results showing that the venom modulates splenic cytokines levels in mice [24].

At the molecular level, the compounds responsible for *Ams*BV-mediated immunomodulation are yet unknown. The exact mechanism is thought to involve melittin, a major peptide component of bee venom, which can modulate immune cells and alter cytokine release patterns [31].

## 4. Conclusions

The venom of the Middle Eastern bee, *Apis mellifera syriaca* (*Ams*BV), has been studied for its potential therapeutic effects on inflammatory conditions and pain modulation. Using a formalin-induced hyperalgesia mice model, we report sex-based variabilities in pain sensitivity changes triggered by formalin. Males’ sensitivity to pain was boosted in our animal model, and efficiently lowered by *Ams*BV injection. The immunomodulatory activity of the venom may be responsible for the pain relief observed in male mice after BV injection. Our findings suggest that bee venom could potentially serve as a therapeutic for pain management, with a tailored approach based on sex differences in immune and hormonal responses. Our study paves the road toward further research to fully understand these mechanisms and optimize bee-venom-based treatments for both male and female subjects.

## 5. Materials and Methods

### 5.1. Venom and Formalin Solutions

*Apis mellifera syriaca* were located in an apiary in Ramlieh, Aley (Lebanon). The venom was collected from a local healthy apiary as described in [24]. *Apis mellifera syriaca* bee venom (*Ams*BV)-lyophilized extract was stored at −20 °C. Venom solution was prepared in phosphate-buffered saline (PBS) and injected at a dose of 1.5 mg/kg. Formalin was prepared from a 40% stock formaldehyde solution that was diluted in PBS to reach a final concentration of 5%.

### 5.2. Mice Handling and Treatment

All animals were handled and experimental procedures were carried out according to the guidelines of the Institutional Animal Care and Use Committee at the University of Balamand, with strict adherence to the ethical guidelines for the study of experimental pain in conscious animals.

Eight- to ten-week-old adult BALB/c mice procured from the University of Balamand animal house were used in this study. Animals (80 males and 80 females) were fed a standard diet and kept at 22 °C in a 12 h day/night cycle and handled according to the Guide for Care and Use of Laboratory Animals of the University of Balamand Faculty of Sciences. Mice were divided into 4 groups, based on the injection received, corresponding to (1) control, (2) formalin, (3) BV, and (4) formalin + BV.

Formalin (5%) was subcutaneously injected into the left hind paw, while BV was injected intraperitoneally. BV was injected 15 min post-formalin injection. The control group (1) was injected with PBS, while group (3) was injected with BV.

### 5.3. Hotplate Test

To assess the thermal pain thresholds, each mouse was placed on a heated pad with the temperature being maintained at T = 51 °C (±0.5 °C). The hotplate test was used to assess the latency of paw licking or jumping, which was taken as an index of the pain threshold [25]. Before injections, each mouse was set on the hotplate and was prepared to have a reaction time of 20 s, to record the baseline of their reaction time prior to any injection. All groups were subjected to the hotplate (HP) test following the injections to record their reaction time at 0.5 hr, 1.5 h, 3.5 h, 24 h, and 48 h.

### 5.4. Dissection

The mice needed for ELISA quantifications were sacrificed by cervical dislocation 1.5 h and 3.5 h post injection. The spleen was collected individually. Each spleen was rinsed with PBS and then dried using filter paper and set in an Eppendorf tube. Once the organs were extracted, snap freezing was used to preserve the organs.

### 5.5. Tissue Homogenization

Collected spleens were individually homogenized using T-10 basic ULTRA-TURRAX for 1 min at 20,000 rpm in 1.2 mL of homogenization buffer, which consisted of 0.2% of NaCl, 0.05% bovine serum albumin (BSA), 0.05% Tween 20, and 2 tablets of protease inhibitor in PBS. The homogenized samples were centrifuged at 14,500 rpm for 15 min at 4 °C; supernatants were removed and stored in pyrogen/endotoxin-free tubes at −20 °C until further usage.

### 5.6. Cytokine Measurement

Quantitative evaluation of cytokines was performed using the Enzyme-Linked Im-muno-Sorbent Assay (ELISA ABTS murine kit, Peprotech, France) according to the manufacturer’s recommendations. Briefly, 100 μL of the prepared supernatants/standards were added into the 96-well plates in triplicates. Plates were read with an ELISA plate reader at 405 nm with 650 nm as the correction wavelength. Concentrations of the cytokines IL-4, IL-6, IL-10, and IFN- γ were estimated using standard curves established with the appropriate recombinant cytokines. The results are expressed as pg/mg of total proteins.

### 5.7. Statistical Analysis

Differences among all groups in all sexes and all timelines were analyzed using GraphPad Prism 6.00 software (GraphPad Software Inc., San Diego, CA, USA) by ANOVA. Results are expressed as means ± SEM. *p* ≤ 0.05 was considered statistically significant.

## Data Availability

The original contributions presented in this study are included in the article. Further inquiries can be directed to the corresponding authors.
